# NRF3 Decreases during Melanoma Carcinogenesis and Is an Independent Prognostic Marker in Melanoma

**DOI:** 10.1155/2022/2240223

**Published:** 2022-03-26

**Authors:** Anni Immonen, Kirsi-Maria Haapasaari, Sini Skarp, Peeter Karihtala, Hanna-Riikka Teppo

**Affiliations:** ^1^Cancer Research and Translational Medicine Research Unit, University of Oulu, Oulu, Finland; ^2^Medical Research Center Oulu, Oulu University Hospital and University of Oulu, Oulu, Finland; ^3^Department of Pathology, Oulu University Hospital, Oulu, Finland; ^4^Infrastructure for Population Studies, Faculty of Medicine, University of Oulu, Oulu, Finland; ^5^Helsinki University Hospital Comprehensive Cancer Center, University of Helsinki and Helsinki University Hospital, Helsinki, Finland

## Abstract

The prognostic significance of the major redox regulator, nuclear factor erythroid-2-related factor 2 (NRF2), is recognized in many cancers, but the role of NRF3 is not studied. Analysis from the Gene Expression Omnibus datasets showed that NRF3 mRNA levels increased from benign to dysplastic naevi (*p* = 0.04). We characterized the immunohistochemical expression of NRF3 in 81 naevi, 67 primary skin melanomas, and 51 lymph node metastases. The immunohistochemical expression of cytoplasmic NRF3 decreased from benign to dysplastic naevi (*p* < 0.001) and further to primary melanomas (*p* < 0.001). High cytoplasmic NRF3 protein expression in pigment cells of the primary melanomas associated with worse melanoma-specific survival in multivariate analysis, specifically in the subgroup of patients with the lymph node metastases at the time of diagnosis (hazard ratio 3.179; 95% confidence interval 1.065-9.493; *p* = 0.038). Intriguingly, we did not observe associations between NRF3 and the traditional prognostic factors such as Breslow thickness, ulceration, or stage. Together, this data represents the primary description about the role of NRF3 in pigment tumours that is worthy of further explorations.

## 1. Introduction

Nuclear factor erythroid-2-like factor 3 (protein as NRF3 and gene as *NFE2L3*) is the least studied member of the Cap'n' collar basic leucine zipper (CNC-bZIP) family of transcription factors, while the roles of NRF1 and especially NRF2 in carcinogenesis have recently been extensively reviewed [1–3]. NRF3 functions in stress-related transcriptional programs to induce the protective response upon several cellular stressors such as oxidative injury and protein aggregation [4]. Compared with a vast overlapping regulation over processes such as cell redox homeostasis, metabolism, cellular movement, and migration mastered by all the three transcription factors, NRF3 appears to regulate the extracellular matrix, protease function, and response to organic nitrogen independently from NRF1 and NRF2 [4]. In contrast to NRF1, the lack of NRF2 or NRF3 does not cause a lethal phenotype in knockout animal models. NRF3 is shown to increase tolerance against proteotoxic stress by activating 20S proteosome assembly during oncogenesis, following inhibition of apoptosis (p53 and Rb), stimulation of the cell cycle, and possibly angiogenesis [4, 5]. Also, NRF3 is shown to play a role in cell migration, invasion, and angiogenesis in pancreatic cancer *in vitro* and has an elevated expression in pancreatic cancers compared to adjacent nonneoplastic tissue [6]. The upregulation of transcriptional and protein expression of NRF3 has also been described in colorectal cancer tissue specimens [7–9]. In contrast to pancreatic and colorectal cancer, NRF3 appears to be downregulated in breast cancer compared to adjacent nonneoplastic breast and have an inhibitory effect on malignant behaviour in breast cancer *in vitro* [10].

Melanoma is a heterogenous cancer type with a broad spectrum of mutational background [11]. Despite recent advances in the molecular subtyping of melanoma and its treatment repertoire [12], the essential nature of this pigment cell cancer is far from being elucidated. Pigment cells are inherently resistant to oxidative stress, and melanoma can exploit the cellular stress responses for its advantage during promotion and progression of the disease [13–15]. We have previously described the expression and prognostic role of NRF2 and NRF1 immunohistochemical expression in primary and metastatic melanoma [16, 17]. Here, we continue to describe the immunohistochemical expression and the prognostic role of NRF3 in a sample set of melanomas, their lymph node metastases, and benign and dysplastic naevi with the addition of NRF3 mRNA expression in three Gene Expression Omnibus (GEO) datasets and The Cancer Genome Atlas sample sets from the TIMER2.0 web server [18].

## 2. Materials and Methods

### 2.1. Patient Samples

The study included 143 patients and 199 patient samples (Table 1) collected from the paraffin block archives stored in the Department of Pathology at Oulu University Hospital between 2001 and 2016. All samples were fixed in neutral-buffered formalin and embedded in paraffin. Cases were randomly collected based on the diagnosis and the adequacy of the samples. The series consisted of 42 benign naevi (20 compositus, 22 intradermal), 39 dysplastic naevi, 30 nodular melanomas, 29 superficially spreading melanomas, and 8 acral melanomas. Out of all malignant samples 32 were metastatic melanomas with, respectively, 51 lymph node metastases available (one or several per case). The median follow-up time of malignant melanomas was 39.0 months. Diagnoses were made according to the current WHO classification [12]. Clinical data and pathologists' reports of the cases were collected retrospectively from the electronic patient records of Oulu University Hospital.

### 2.2. GEO Datasets

The three microarray datasets GSE8401, GSE46517, and GSE53223 first described in original articles [19–21] were preprocessed and analyzed as previously described [17]. Data was analyzed using Chipster v3.14 software [22]. The differential mRNA expression level of NRF3 was determined and tested with the empirical Bayes *t*-test between the diagnostical groups. The combined data contained normal skin samples (*n* = 13), benign (*n* = 14) and dysplastic naevi (*n* = 7), primary melanoma lesions (*n* = 62), and metastatic melanoma lesions (*n* = 104). The respective results were plotted with GraphPad Prism 8.

### 2.3. Cell Lines

Cell lines representing normal human epidermal melanocytes (C-12402, PromoCell GmbH, Germany), human primary melanoma IPC-298 (ACC 251), and metastatic melanomas SK-MEL-30 (ACC 151) and COLO-800 (ACC 193) were ordered from Leibniz-Institut, DSMZ (Braunschweig, Germany). Melanocytes were cultured in Melanocyte Growth Medium M2 (C-24300, PromoCell), and melanoma cells were cultured in RPMI-1640 (R8758, Sigma-Aldrich) with 10% foetal bovine serum and 100 IU/ml penicillin and streptomycin (Pen-Strep solution, HyClone Laboratories, Inc. UT, USA). Cells were cultured at 37°C 5% CO_2_.

### 2.4. NRF3 Immunohistochemistry

Sections of 3.5 *μ*m thickness were cut from samples routinely fixed in formalin and embedded in paraffin. Batches of 40 samples were hand processed per one staining procedure. Skin adnexal structures (sweat glands and apocrine glands) and vasculature served as the positive control between the batches. A negative control without a primary antibody was prepared. Tissue sections were deparaffinised in xylene (3 min, 3 times) and rehydrated through graded ethanol. For the NRF3 antibody (HPA055889 Sigma-Aldrich, Merck, Darmstadt, Germany) staining, antigen retrieval was performed with TrisEDTA pH 9 by boiling with microwaves at 850 W for 2 minutes and 150 W for 15 minutes. After boiling, the sections were allowed to cool at room temperature (RT) and washed using distilled water and PBS-Tween 20. The sections were incubated in peroxidase blocking solution for 5 minutes to inactivate endogenous peroxidases. After washing twice with PBS-Tween 20 for 5 minutes, sections were incubated with the primary antibody for 60 minutes at RT with 1 : 100 dilution, then washed repeatedly with PBS-TWEEN for 5 minutes, and incubated with a secondary antibody and visualized with DAB according to the manufacturer's instructions (DAKO EnVision, Agilent K5007, CA, USA). After being rinsed with distilled water, sections were counterstained with haematoxylin, rinsed, dehydrated, cleared, and mounted. To evaluate the immunohistochemical data, the staining intensity was evaluated from the tumorous cells as one of the following expressions: negative (0), weak positive (1), or strong positive (2). The quantity of each intensity level was recorded (0-100%). Subsequently, a modified histoscore was used with the following algorithm: 0 × negative expression percentage + 1 × weak expression percentage + 2 × strong expression percentage (range 0-200).

### 2.5. Western Blot Analysis

The whole cell lysates were prepared by using RIPA Lysis and Extraction Buffer (Thermo Scientific, IL, USA) with Pierce Protease and Phosphatase Inhibitor Mini (Thermo Scientific, IL, USA). The fractionated lysates were prepared by using the Subcellular Protein Fractionation Kit for Cultured Cells (Thermo Scientific, IL, USA). Protein concentrations were measured using the Bio-Rad Protein Assay (Bio-Rad; CA, USA), and the concentration in individual samples was equalized before adding 4x Laemmli buffer to a final concentration of 1x. Equal amounts of protein (50 *μ*g whole cell lysates, 45 *μ*g fractionated lysates) were run on 10% SDS-PAGE gels and then transferred with iBlot 2 Transfer Stacks and iBlot 2 Dry Blotting System onto PVDF membranes (Invitrogen, Thermo Scientific, IL, USA). After one hour 5% milk block, membranes were probed with the antibodies diluted with 5% BSA (NRF3) or 5% milk (*β*-actin and PCNA and secondary antibodies) in Tris-buffered saline with 0.1% Tween 20. Primary antibodies anti-NRF3 (HPA055889, Sigma-Aldrich), anti-*β*-actin (NB600-501, Novus Biologicals, UK), and anti-PCNA (NB600-501SS, Novus Biologicals, UK) were incubated overnight, and appropriate HRP-conjugated secondary antibodies (sc-2054 and sc-2055, Santa Cruz, CA, USA) were incubated at RT for one hour. Blots were detected with the Western blot imaging system Azure 600 (Azure Biosystems, CA, USA).

### 2.6. Statistical Analyses

Statistical analyses were performed by using IBM SPSS Statistics software, v. 26.0.0.0 (IBM Corporation, Armonk, NY, USA). The significance of associations was defined by using the Mann–Whitney *U* test, Kruskal-Wallis test, paired *t*-test, and Spearman's rho test with correlation coefficient. Kaplan-Meier curves with the log-rank test were applied in survival analyses, along with Cox regression to perform multivariate analysis. The patients with distant metastases at the time of diagnosis were excluded from the survival analyses. In determining a two-classed variable for survival analysis, a histoscore cut-off value (110) was found as an optimal cut-off for NRF3. Melanoma-specific survival was calculated from the time of diagnosis to the time of confirmed melanoma-related death. Values of 𝑝 of less than 0.05 were considered significant.

### 2.7. Ethical Approval

The study was approved by the Finnish National Supervisory Authority for Welfare and Health and the Local Ethics Committee of the Northern Ostrobothnia Hospital District. During data collection and management, the principles of the Helsinki Declaration were followed. The authors declare that they have no competing interests and that funding sources had no involvement in the study.

## 3. Results

### 3.1. mRNA Expression Based on GEO Data and TIMER2.0

**Figure 1:**
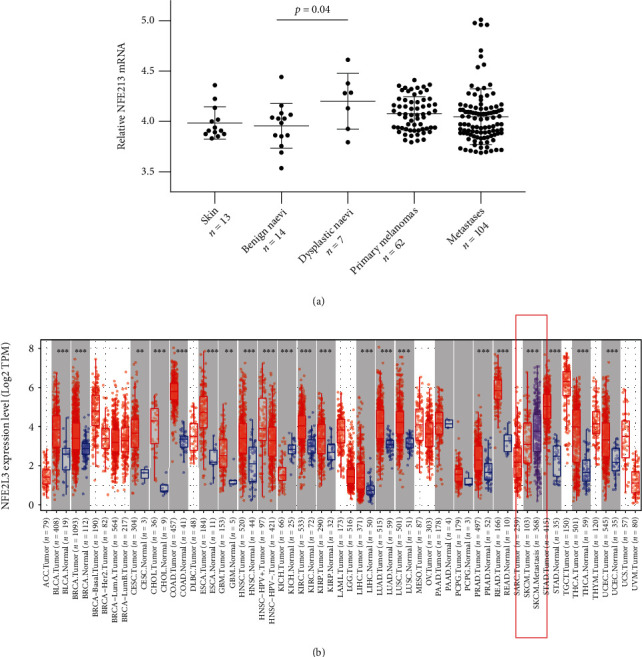
mRNA expression. (a) Pooled Gene Expression Omnibus data from three different cDNA microarray studies including the expression levels of NRF3 show varying levels between benign, dysplastic, and malignant conditions. The only observed statistical significance is indicated. (b) A TIMER2.0 expression plot based on TCGA database sample sets [18]. Skin cutaneous melanoma and melanoma metastasis groups are highlighted with a red box. NFE2L3 mRNA levels increase significantly between melanoma tumour and metastasis groups (^∗∗∗^*p* value < 0.001).

We did not observe a significant difference in NRF3 mRNA levels between normal skin samples and benign naevi. The level of NRF3 mRNA increased significantly between benign and dysplastic naevi (*p* = 0.04), but no difference was seen between dysplastic naevi and primary melanoma or melanoma and metastasis (Figure 1(a)). To complement these results, a significant increase in NRF3 mRNA levels between primary (*n* = 103) and metastatic cutaneous melanomas (*n* = 368) was seen in the TIMER2.0 [18] The Cancer Genome Atlas sample set (*p* value < 0.001, Figure 1(b)).

### 3.2. Western Blotting and Immunohistochemical Expression of NRF3 in Naevi, Primary Melanomas, and Melanoma Metastases and Their Association with Histopathological and Clinical Parameters

**Figure 2:**
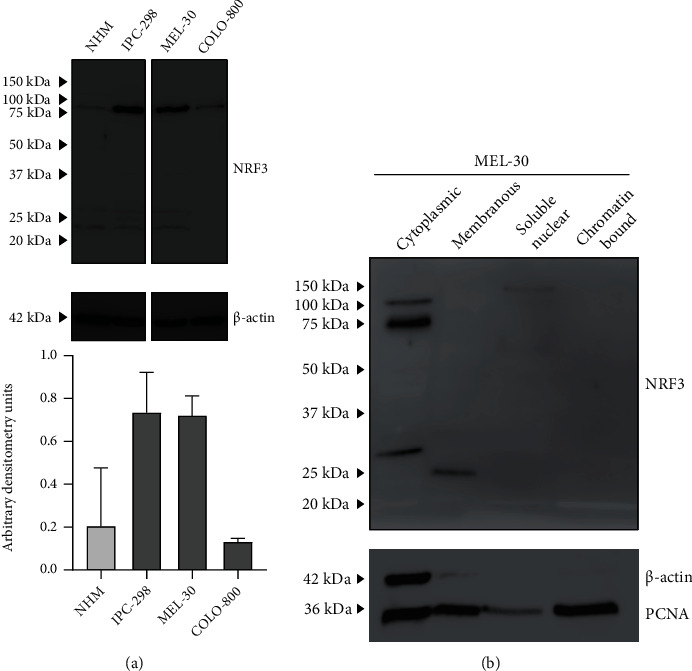
Protein expression of NRF3 in Western blot. (a) Whole cell lysates from normal human melanocytes (NHM) and IPC-298, MEL-30, and COLO-800 melanoma cells. *β*-Actin serves as the loading control. NRF3 expression is detected in a molecular weight of approximately 80-90 kDa. (b) Fractionated lysate from MEL-30. *β*-Actin and PCNA serve as loading controls. Nrf3 expression is located mainly in the cytoplasmic fraction.

In Western blot analysis, the NRF3 protein was detected in whole cell lysates of normal human melanocytes, primary melanomas, and metastatic melanoma cells (Figure 2(a)). NRF3 expression was detected in a molecular weight of approximately 80-90 kDa and more intensively in primary and metastatic melanoma cell lines IPC-298 and MEL-30 compared to normal human melanocytes and the metastatic melanoma cell line COLO-800. Also, bands of smaller size, near 25 kDa, were present. In fractionated lysates from MEL-30, NRF3 was mainly detected in the cytoplasmic fraction (Figure 2(b)).

**Figure 3:**
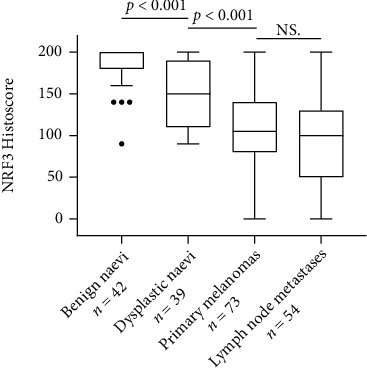
A boxplot diagram representing the modified histoscore of the immunohistochemical expression of NRF3 in paraffin-embedded patient samples. A significant decrease in expression was seen between benign and dysplastic naevi and between dysplastic naevi and primary melanomas. NS = no statistical significance.

**Figure 4:**
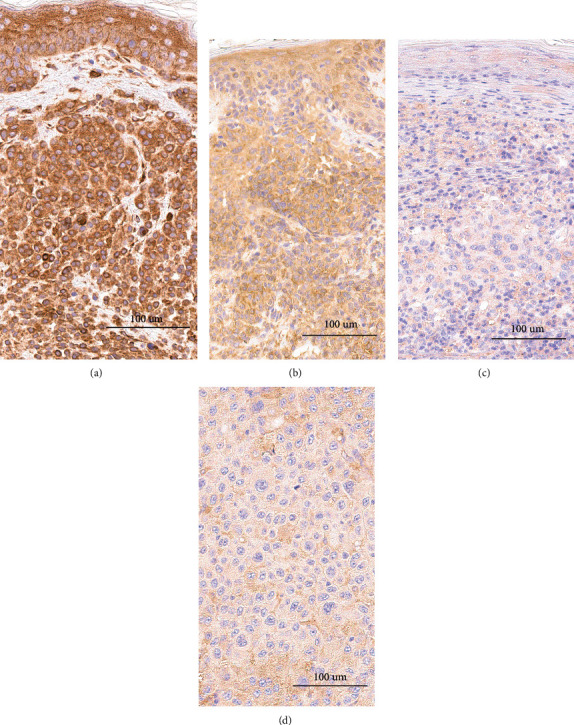
Immunohistochemical expression of NRF3 in benign naevus (a), dysplastic naevus (b), primary melanoma (c), and metastatic melanoma from a lymph node (d). The expression level is decreasing from benign to dysplastic and malignant samples.

Immunopositivity of NRF3 was detected only in the cytoplasm. The expression decreased from benign to dysplastic naevi (*p* < 0.001, Figures 3, 4(a), and 4(b)) and then further to primary melanomas (*p* < 0.001, Figures 3, 4(b), and 4(c)) but remained then at the same level in metastases (Figures 3, 4(c), and 4(d)). We observed no difference between NRF3 expression in primary melanomas and corresponding metastases (paired *t*-test *p* = 0.17). The immunohistochemical expression of NRF3 was not associated with Breslow's thickness, Clark level, ulceration, mitotic activity, tumour-infiltrating lymphocytes, pigmentation, histological type of melanoma, melanoma patients' age, gender, lesion location, or nodal status (Table 2).

### 3.3. Survival and Cox Regression Analysis

**Figure 5:**
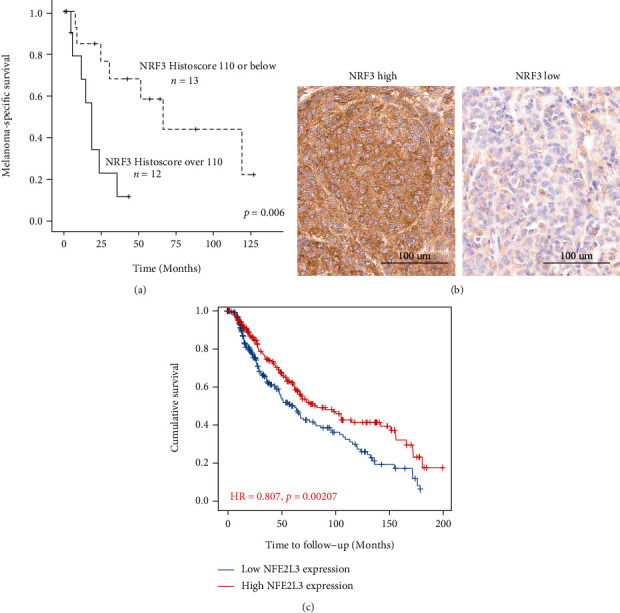
(a) Kaplan-Meier (*n* = 25). High cytoplasmic NRF3 expression (cut-off 110) is associated with worse melanoma-specific survival within a group of patients with nodal metastases at the time of diagnosis. (b) Primary melanoma cases with high and low NRF3 expression. (c) A TIMER2.0 Kaplan-Meier plot based on TCGA database sample sets [18]. NFE2L3 mRNA expression in primary and metastatic melanomas (*n* = 471). Low expressors have a worse survival.

A high cytoplasmic NRF3 immunohistochemical expression in melanoma cells in primary melanoma samples correlated with a worse melanoma-specific survival (log-rank test *p* = 0.006), but only in the patients with nodal metastases at the time of diagnosis (*n* = 25, Figure 5(a)). Kaplan-Meier estimates for median survival in the patients with nodal metastases at the time of diagnosis were 19.0 months in those with high NRF3 expression and 67.0 months for those with low NRF3 expression. The expression of NRF3 in the lymph nodes was not associated with prognosis of these node-positive patients. In patients without nodal metastases at the time of diagnosis (*n* = 36), the immunohistochemical expression of NRF3 had no prognostic significance in the log-rank test.  Figure 5(b) demonstrates the notable variability of immunohistochemical expression of NRF3 in primary melanoma samples.

In multivariate analysis, the immunohistochemical expression of NRF3 in the cytoplasm of melanoma cells was a more significant predictor of poor melanoma-specific survival than Breslow thickness, but again only in the patients with nodal metastases at the time of diagnosis (for NRF3: hazard ratio (HR) 3.179, 95% confidence interval (CI) 1.065-9.493, *p* = 0.038, and for Breslow: HR 1.021, 95% CI 0.978-1.067, *p* = 0.345, respectively). Instead, in node-negative patients, NRF3 was not observed to be a significant predictor of survival (for NRF3: HR 0.546, 95% CI 0.109-2.743, *p* = 0.462, and for Breslow: HR 1.431, 95% CI 1.053-1.945, *p* = 0.022, respectively).

Analyzed from the TIMER2.0 database [18], the cases with low *NFE2L3* mRNA expression had a worse survival compared to those with high mRNA expression in primary and metastatic melanoma patients (Figure 5(c)).

## 4. Discussion

In this work, we studied for the first time the transcriptional levels and protein expression of the redox-sensitive transcription factor NRF3 in melanoma. The transcriptional levels were studied using three GEO microarray data patient sample sets merged. The differences in transcriptional levels of NRF3 between diagnostic groups were minor showing a significant increase only in dysplastic naevi compared to benign naevi. However, a significant increase in NRF3 mRNA levels between primary and metastatic cutaneous melanoma sample sets was seen in TCGA. An antibody detecting the NRF3 protein was expressed in melanocyte and melanoma cell lines with mainly cytoplasmic expression in Western blotting. Immunohistochemical expression of NRF3 was then studied in a sample set of benign and dysplastic naevi, melanomas, and their lymph node metastases showing that similar to NRF2 and NRF1 [17], NRF3 was also downregulated during melanoma carcinogenesis at the protein level. In the patients with lymph node metastases at the time of diagnosis, high NRF3 protein expressors had worse survival, independently from Breslow's thickness. In contrast, low mRNA levels were associated with worse survival in melanoma in the TIMER2.0 database.

### 4.1. Transcriptional and Protein Expression of NRF3

The mRNA levels of NRF3 in different malignancies are mainly upregulated in tumoural tissue compared to normal counterparts [23]. To our knowledge, the only reported exception to this is chromophobe renal cell carcinoma. Upregulation of transcriptional levels could be explained epigenetically, since hypomethylation of the *NFE2L3* gene is associated with higher *NFE2L3* mRNA levels in renal clear cell carcinoma [24]. Similar to the majority of different cancer types, we demonstrated the increasing trend of *NFE2L3* mRNA levels from benign to dysplastic naevi. This change might indicate that NRF3 can be deregulated in the premalignant phase of melanoma development with cumulative oncogenic mutations between benign and dysplastic naevi [25]. NRF3 is shown to have a proapoptotic function in basal keratinocytes provoked with ultraviolet B in an experimental setting [26], but similar data on NRF3 in pigment cells is virtually lacking [27]. The pooled GEO dataset should be interpreted with caution, as the normal skin and premalignant samples are scant in number. Possibly due to a small sample size, the GEO dataset was unable to show significant changes of transcriptional levels between primary melanoma and metastases, while the data from TCGA samples indicated that mRNA levels increased significantly from primary to metastatic melanoma samples. Also, TCGA material showed a large variance in *NFE2L3* expression levels in both primary and metastatic melanoma with overlapping quartiles, and therefore, a large set of samples would be needed to recognize a significant trend.

The affinity purified antibody used against NRF3 was chosen based on the Human Protein Atlas database (THPA) [28]. The antibody target site is specific for the product of longer protein coding transcript of *NFE2L3* and specific only to NRF3 of the NFE2L family [29]. Intriguingly, we observed NRF3 expression only in the cytoplasm, but not in the nucleus, similarly to original studies using the same antibody [6, 10, 26]. Immunohistochemical observation is supported by fractionated Western blot that showed no significant nuclear signal with the same antibody. The authenticity of NRF3 antibodies is discussed in a review by Kobayashi [5], and the suitability of the used antibody for Western blotting is supported in THPA web pages [28]. As NRF3 is processed posttranscriptionally and requires cleavage from the endoplasmic reticulum membrane [30], we assume that possible alterations in protein configuration or interactions with regulatory partners could affect the epitope region availability in the nucleus. Unfortunately, we were unable to visualize NRF3 in immunoelectron microscopy with this antibody.

The majority of cancer types evaluating tissue specimens have shown an increase in NRF3 protein expression in malignant compared to more benign tissues. For example, in colorectal carcinoma [7, 8], protein expression was increased in tumour resection samples when set against with the adjacent mucosa. Contradicting these, a report from Zhou et al. [31] showed a decrease in NRF3 expression in colorectal carcinoma compared to adjacent normal tissue, but this study used a different antibody than most of the studies. Further on, in a study from Wang et al., NRF3 protein expression was significantly increased in pancreatic cancer tissue in comparison with adjacent nontumour tissue [6]. In contrast, in breast cancer tissue, NRF3 expression was suppressed compared with that in benign breast tissue [10]. This is similar to our observation in our melanoma sample set showing the decrease in NRF3 protein expression from benign to dysplastic naevi and further to primary melanomas.

### 4.2. The Prognostic Significance of NRF3

By browsing *NFE2L3* mRNA expression data and survival data from the public database TIMER2.0 [18], high mRNA expression can be associated with either poor or better prognosis variably in different malignancies. In the case of melanoma, low *NFE2L3* mRNA expressors had a worse prognosis. High mRNA and protein levels of NRF3 are associated with advanced TNM stages with a poor survival in pancreatic cancer [6], and high *NFE2L3* mRNA is associated with a higher grade and stage in hepatocellular carcinoma [32]. High NRF3 protein expression is associates with a higher stage in colorectal cancer [8] but again shows discrepancy with Zhou et al. [31] where high protein expression favours better prognosis. Based on integrative bioinformatics, *NFE2L3* has been considered one of the nine prognostic genes in colorectal cancer predicting improved overall survival [33]. In our material, NRF3 expression was not associated with any tested clinical or pathological parameters or traditional prognostic factors of melanoma, but the cases retaining high NRF3 protein expression in their primary tumours had a poorer melanoma-specific survival compared to low expressors. This observation was found only in the patients with lymph node metastases at the time of diagnosis. This is clinically a highly relevant subgroup, due to their poor prognosis [34]. Considering that NRF3 is an independent regulator of cell-matrix interactions, proteasome activity, apoptosis, and proliferation [26, 30, 35], the expression and function of NRF3 can have a crucial impact on melanoma carcinogenesis and metastatic potential. In our material, the patients with lymph node metastases and high NRF3 expression demonstrated an estimated median of melanoma-specific survival of only 19.0 months. This is even more striking when set against the background that all patients were originally treated with a surgery with a curative intention and suggests not only rapid dissemination of the disease but also the presence of severe therapy resistance in the metastatic setting. It is still worth noting that the majority of these melanoma patients were treated before the era of immuno-oncological and targeted treatments.

Here, we described the increasing transcriptional level of *NFE2L3* and decreasing protein level of NRF3 in melanoma carcinogenesis while poor survival is associated with high mRNA and protein levels of NRF3. The biology of NRF3 is far from being understood, but what is evident is the high variance of the posttranslational structure and the complexity of the regulation of NRF3 and its aberration in cancer [5, 7, 30, 36, 37]. All these factors can affect the recognition of the protein with any used antibodies and explain the discrepancy between mRNA and protein levels. Also, the posttranscriptional modulation of gene expression by microRNAs, which is most commonly silencing or degradation of target mRNA [38], can explain the discordance between transcriptional and protein expression levels and also the variance between different cancer types. A possible miRNA behind the observed increasing level of *NFE2L3* mRNA and decreasing protein level of NRF3 could be miR-1246 that is increased in melanoma compared to normal tissues [39] and targets *NFE2L3* as a negative regulator (40). Then, cases with high mRNA and protein levels and associated poor prognosis could be then explained with lower level of miR-1246. However, the fundamental mechanisms behind the close relationship between dismal prognosis and high NRF3 expression were not in the primary scope of this study. Finally, these observations were made from separate publicly available datasets and our FFPE patient samples, and so the apparent discrepancy of transcriptional and protein levels should be reconsidered from a same sample set in the future. As a potential drawback, this study was retrospective in nature with a relatively small sample size in each cohort. Nevertheless, the importance of NRF3 also in melanoma should be noted, and its role confirmed in experimental settings and therapeutic prospects should be explored.

## 5. Conclusions

This data suggests that there is a clear loss of NRF3 protein expression during different stages of melanoma carcinogenesis, while mRNA levels increase in dysplastic naevi and later in the metastatic phase. This discrepancy may be explained at least by the posttranscriptional modulation of NRF3. Retaining high cytoplasmic NRF3 protein expression predicts a dismal outcome in patients with nodal metastases, even more significantly than Breslow thickness, the most powerful prognostic factor of cutaneous melanoma. Thus, it is plausible that even if NRF3 would have a protective role against melanoma carcinogenesis, it seems to be exploited as a tumour progressing factor in the malignant phase, as suggested earlier in some other tumour types.

## Figures and Tables

**Table 1:** Patient cohort.

	*n*
Total number of patients	143
Age median (years)	60
Samples per diagnosis	
Compound naevus	20
Intradermal naevus	22
Dysplastic naevus	39
Nodular melanoma	30
Superficially spreading melanoma	29
Acral melanoma	8
Metastasis	51
Number of patients with malignant melanoma	63
Median age (years)	70
Gender	
Males	44
Females	19
Breslow's thickness	
≤1 mm	11
1–1.9 mm	14
2–3.9 mm	13
>4 mm	29
Breslow mean (mm)	4.98
Breslow median (mm)	3.50
Clark level	
I-II	2
III-V	65
Ulceration	
Yes	26
No ulceration (one case not defined)	41
Mitotic activity	
Low	38
High	29
Tumour-infiltrating lymphocytes	
Absence	28
Nonbrisk	30
Brisk	9
Pigmentation	
None to average	42
Abundant	25
Melanoma location	
Head or neck	19
Upper limb	10
Body	24
Lower limb	14
N	
0	38
1	8
2	11
3	10

**Table 2:** Correlation of NRF3 immunohistochemistry in primary melanomas with pathologic and clinical variables.

	Correlation with NRF3 histoscore, *p* value	Cases tested (*n*)
Breslow's thickness	0.794	67
Clark level	0.791	67
Ulceration	0.519	67
Mitotic activity	0.511	67
Tumour-infiltrating lymphocytes	0.207	67
Pigmentation	0.176	67
Histological type of melanoma	0.395	67
Age	0.853	63
Gender	0.230	63
Lesion location	0.750	67
Nodal status	0.564	67

## Data Availability

The data used to support the findings of this study are available from the corresponding author upon request.
